# Same Gender Erotomania: When the Psychiatrist Became the Delusional Theme—A Case Report and Literature Review

**DOI:** 10.1155/2021/7463272

**Published:** 2021-09-01

**Authors:** Ruzita Jamaluddin

**Affiliations:** ^1^Department of Psychiatry, Hospital Tuanku Fauziah, Perlis, Ministry of Health Malaysia, Malaysia; ^2^Clinical Research Centre, Hospital Tuanku Fauziah, Perlis, Ministry of Health Malaysia, Malaysia

## Abstract

Erotomania is a rare subtype of delusional disorder, whereby the affected person believes that another individual, usually someone with higher socio-economic status, is in love with them despite having little or no contact. To the best of our knowledge, this is the first published case of same gender erotomania, involving a 28-year-old single lady and a 42-year-old divorcee towards a female psychiatrist. We aimed to share the challenges experienced by the managing psychiatrist as she inopportunely became the theme of her patients' delusion. We also reviewed and discussed recent literature on erotomania to create awareness among psychiatrists towards this rare psychiatric condition.

## 1. Introduction

The incidence of delusional disorder is rare, estimated to be around 1 to 3 cases per 100,000 population [1], with erotomania-subtype being much rarer [2]. Erotomania refers to the persistent delusional belief that another person, usually of someone with higher socio-economic status, is in love with the affected person, despite having little or no contact [3, 4].

Herein, we report two unique cases of same gender erotomania involving a 28-year-old single lady and a 42-year-old divorcee towards a female psychiatrist in the setting of a therapeutic relationship. We aimed to share the challenges experienced by the managing psychiatrist as she became the theme of her patients' delusion. Our writing also discusses relevant literatures on the prevalence, risk factors, and clinical approach in erotomania cases to create awareness towards this rare psychiatric condition.

## 2. Case 1

The first case described a 28-year-old lady who was first reviewed by a psychiatrist at the age of 18-year-old for suspected conversion symptoms when she presented with right-sided limb weakness following a trivial fall. She further narrated that she did not feel loved and was not treated equally among her siblings by the parents. She admitted having some depressive symptoms but does not fulfill criteria for major depressive disorder. At that point, she was diagnosed as having dysthymic disorder with conversion symptoms. Psychoeducation, reassurance, and supportive psychotherapy were given during the hour-long consultation. No oral medication was prescribed. She was given an open appointment for psychiatric evaluation at the nearest hospital due to logistic reason. The session was the only encounter with the psychiatrist.

However, since the first meeting, the patient had believed that something truly romantic had occurred between them. She had been following the psychiatrist through Facebook™ and made several picture collages of the psychiatrist, recovered from the therapist's personal social media account. She had even created multiple fake Facebook™, WeChat™, and Twitter™ accounts in the psychiatrist's name and had posted several affectionate videos to manifest the romantic love she had for the psychiatrist. She even succeeded to retrieve the psychiatrist's private phone number from the office, after impersonating as a family member. Since then, the psychiatrist had also been receiving regular missed phone calls, letters, and text messages from more than 10 different mobile numbers on a weekly basis.

Despite being advised against the stalking behavior by the psychiatrist, she continued to text the psychiatrist and mostly vents about her life struggles and how she did not feel loved and wanted. Gradually, the content became threatening as she sought for more attention, claiming that her condition was deteriorating with frequent auditory hallucinations and had self-injurious behavior, but persistently refused to attend the clinic for further evaluation and treatment. She also claimed that she has been physically abused by the parents. Therefore, the local welfare department was alerted to further investigate and perform home visit; however, they affirmed that the patient has had two accounts of making bogus calls and fabricating abuse stories to the National Urgent Response (NUR), which was the hotline number set up by the Malaysian Ministry of Women, Family and Community Development (MWFCD) to enable early intervention for victims of domestic violence. The cases were eventually dismissed after a closed session with the parents and careful medical examination. Apart from that, several psychosocial investigations were performed involving the schoolteachers and schoolmates which revealed no impairment in academic achievement or social functioning. The parents were regularly updated on the patient progress and informed of the need for psychiatric evaluation, but unfortunately, they dismissed the issue as frivolous. However, once police report was lodged by the psychiatrist of the perpetual physical harassment of the patient trailing her at work, they eventually agreed for psychiatric evaluation by a different psychiatrist.

Her mental status examination revealed a fixed and systematized delusions that the first therapist was in love with her since the first consultation and that the love was mutual. Her affect was appropriate, and her speech was relevant and coherent. She denied delusion of reference or control and denied of having sexual fantasies with the therapist. Her cognitive functions were intact except for her poor insight. No mood symptoms were elicited. Urine drugs were negative, and routine blood investigations were normal.

She was the second out of five siblings. Her parents did not notice any behavioral changes at home and denied any traumatic events and physical or sexual abuse throughout the patient's childhood. The patient denied of having past romantic relationships. The parents, however, admitted that the patient was commonly cared for by a babysitter during her early childhood as both parents were occupied with their career development.

Since then, she had been seen by three different psychiatrists, and the diagnosis remained as primary erotomania towards the first psychiatrist. She was initially prescribed with oral antipsychotics, but due to her poor insight and poor oral compliance, she was decided for monthly intramuscular depot injection. With ongoing treatment, she had ceased any form of physical contact with the first psychiatrist but remained convinced that the first psychiatrist was in love with her.

## 3. Case 2

The second case described a 42-year-old lady, a divorcee diagnosed with schizophrenia at the age of 32. She came from a lower socio-economic background with poor social support. Previously, she had experienced physical and psychological abuse by her ex-husband who was an alcoholic. She had been shunned away by her own family and had to surrender the custody of her 9-year-old son following the divorce due to her mental illness.

She has had few suicidal attempts by overdosing paracetamol due to marital conflict. With regard to her psychiatric illness, she poorly complies to her oral medication and had multiple hospital readmissions due to relapse psychotic symptoms.

She developed erotomanic delusion when she first met a female therapist three years after her first diagnosis of schizophrenia. She truly believed that the therapist was in love with her following their first meeting. Apart from having delusion of control and paranoid delusion, she also experienced third-person auditory hallucination and tactile hallucination of having sexual intercourse with the therapist. She had bizarre delusion, likening her therapist to a God, and that the therapist was destined to be her soulmate. She was convinced that they will be together when the therapist is reincarnated as a male in her next life. The patient habitually sent love letters, elaborate gifts, and candies to the therapist, including lavish flower bouquets on Valentines. She also developed stalker-like behavior, peeking the therapist at work. In the attempt to persuade the patient for depot antipsychotic injections, the therapist had promised to continue to see the patient for subsequent routine consultation.

She responded well with intramuscular fluphenazine 37.5 mg monthly with improvement of psychotic symptoms, including erotomania. Unfortunately, once she was clinically well, she began to default treatment and follow-up causing relapse psychotic symptoms.

## 4. Discussion

These two cases underscore the challenges of the managing psychiatrist of erotomanic patients when she became the theme of her patients' delusions; the first case exemplifies primary erotomania with borderline personality disorder, whilst the second was a case of schizophrenia with delusion of erotomania. In the modern age of technology where social media mediates social cognizance [5], invasion and breach of privacy and personal space have become easy target for public access, hence a frequent venue to spur stalking behavior.

Problematic behaviors exhibited by the patients, such as incessant calling, sending continuous letters and gifts, and persistent stalking behaviors risk psychological stress to the managing psychiatrist. Maintaining professional relationship in such cases proves to be a challenge as the responsibility to treat is easily tipped with annoyance and frustration. Transfer of care may appear as the likely escape, but risk compliance issues and eventual poor treatment outcome.

Erotomania is a rare type of delusional disorder, moreover with same gender erotomania. Our literature review was conducted using *Web of Science* and *PubMed* using specific sets of keywords: “erotomania,” “de Clerambault's syndrome,” and “old maid's insanity.” Articles published in English in the last three years from 1 January 2019 to date were included for synthesis and review. This yielded a total of 30 articles with 7 duplicates. There were no papers with regard to same gender erotomania as exhibited in our cases.

Erotomania may either present as a primary mental disorder or in association to other psychiatric illness, such as with bipolar I disorder or schizophrenia. Primary erotomania, or otherwise referred to as de Clerambault's syndrome or Old Maid's Insanity, may exist without comorbidities.

Primary erotomania may present with a sudden onset and leads a chronic course, presumed to result from a coping mechanism from severe loneliness following a major loss [6, 7] whereas secondary erotomania is associated with other mental disorders [8] and has a more gradual onset. Patients with erotomania were also found to have structural brain abnormalities [9, 10] such as heightened temporal lobe asymmetry with increased volumes of lateral ventricles.

The ideal treatment for patients with erotomania should be tailored on a case-by-case basis [11]. Risk of symptom relapses is higher due to presence of psychosocial stressors with poor medication adherence. Nonpharmacologic treatments [12] and structured risk assessment are very important [13].

## Data Availability

The datasets associated with this paper are embedded within the text.
